# Specific Preoperative Dynamic Contrast-Enhanced MRI Semi-quantitative Markers Can Correlate With Vascularity in Specific Areas of Glioblastoma Tissue and Predict Recurrence

**DOI:** 10.7759/cureus.15528

**Published:** 2021-06-08

**Authors:** Mohammed A Azab, Sherief Ghozy, Sherif F Hassanein, Ahmed Y Azzam

**Affiliations:** 1 Department of Biochemistry, Boise State University, Boise, USA; 2 Neurological Surgery, Cairo University Hospital, Cairo, EGY; 3 Neurological Surgery, Faculty of Medicine, Mansoura University, Mansoura, EGY; 4 Ophthalmology, Faculty of Medicine, Cairo University, Cairo, EGY; 5 Neurological Surgery, October 6 University, 6th of October City, EGY

**Keywords:** brain tumours, dce, mri, glioblastoma, glioma

## Abstract

Background: Glioblastoma is one of the most aggressive tumours despite all advanced therapies. We aimed to investigate the correlation between qualitative markers of dynamic contrast-enhanced magnetic resonance imaging and vascularity in different tumour regions and elucidate their potential in predicting recurrence.

Methods: Radiological markers of vascularity as wash-in rate, washout rate, and capillary time to peak in different single tumour regions were extracted for all glioblastoma patients before being surgically resected using preoperative dynamic contrast-enhanced MRI (DCE-MRI). Tissue samples were obtained from different intratumoral regions and peritumoral oedema and evaluated for the vascular endothelial growth factor (VEGF).

Results: Two hundred sixty individuals were included in the final analysis, with 180 dead ones and 80 survivors. Radio- and chemo-therapy were received by all surviving patients and 77.8% (n= 140) of the dead ones. The mean time to peak, in seconds, was longest at the peritumoral oedema region (71.7±23.5), followed by the tumour's necrotic centre (50.0±28.5) and its periphery (2.9±1.8). The expression of VEGF at the peritumoral edema region was inversely correlated to the washout rate at the periphery (r= -0.66; P-value= 0.014) and positively correlated to peritumoral TTP (r= 0.94; P-value< 0.001).

Conclusion: Using DCE-MRI, VEGF expression may be used as a non-invasive marker to estimate tumour grade for clinical diagnosis and treatment. Moreover, the risk of glioblastoma recurrence could be determined by evaluating the washout rate at the tumour's periphery. Further large-scale studies are needed to validate the results and to have concrete evidence.

## Introduction

Glioblastoma (GBM) is considered to be the most aggressive primary brain tumour with a horrible prognosis. Recurrence after treatment is a significant problem. The survival rate for one year is about 39.7% [1]. Ideal outcomes are still challenging to be achieved despite recent treatment combinations. The ultimate capacity to regrow after resection is due to the availability of self-regenerating stem cell populations. The biology of GBM is complex and involves many signalling pathways.

One of the most striking features of GBM is hypoxia. GBM is a highly vascularized tumour with characteristically aberrant vessels [2]. Pseudopalisading, areas of necrosis and abnormal capillary growth are typical microscopic features of GBM [3]. Tumour hypoxia is a critical biological feature that affects the response of GBM to different therapies [4]. Since Folkman tried to expose the biological aspect of tumour angiogenesis, several advances have been made [5]. Hypoxia plays an essential role in gene regulation, stem cell recapitulation, and tumour vascularization [6]. Conventional MRI techniques provide details about the anatomy and gross morphology of cerebral lesions, but they fail to unveil the physiological changes at the microcapillary level. Recent MRI techniques can capture certain parameters based on physiological tumour features [7]. Conventional anatomical MRI is sometimes fallacious in the preoperative prediction of tumour pathological grade and vascularity, impacting treatment strategy [8]. Dynamic contrast-enhanced MRI (DCE-MRI) can quantitatively and qualitatively evaluate the hemodynamic changes as blood flow and vascularity at the tissue level [9]. The measured parameters are considered markers for tissue hypoxia.

The maestro regulator of hypoxia-induced changes is the HIF gene [10]. Several tissue markers of hypoxia are identified as vascular endothelial growth factor (VEGF), glucose transporter-1 (GLUT-1), and carbonic anhydrase [11,12]. There is a degree of correlation between tumour vascularity, pathological grade, and hypoxia. We did not find any association between the most prominent vascular marker VEGF expression and GBM recurrence, although some studies reported a correlation [13,14]. That may be because the sampling of the tumour’s different regions was random and not fully representative. We hypothesize that the measured radiological markers may correlate with pathological markers in different areas of GBM. They may also have a role in predicting recurrence.

## Materials and methods

All methods were carried out following relevant guidelines and regulations. The ethical board approved all experimental protocols used in this study. Informed consent was obtained from all subjects or a parent/legal guardian if under 18 years of age. In the case of dead patients, the legal guardian consented.

Patient enrollment

**Figure 1:**
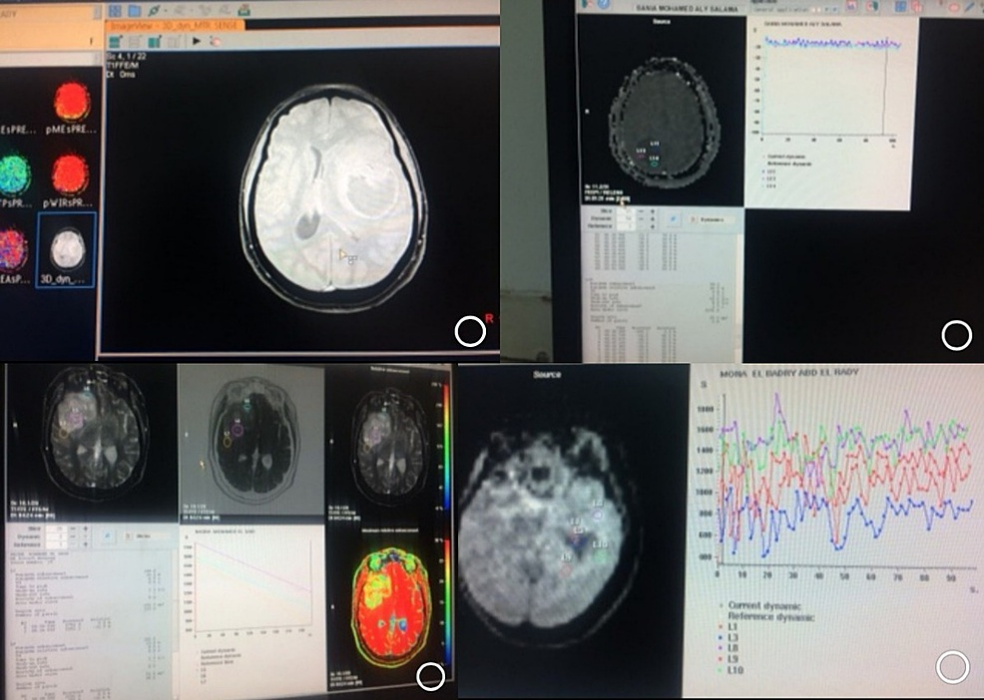
DCE workstation at the enrollment center

This is a prospective single-centre study. Patients with a history of newly diagnosed GBM with a maximum diameter from 3 to 5 cm as a single lesion identified by MRI were enrolled (Figure 1). The lesions were surgically resected at the time of the presentation. The survival status of the patients was tracked from the follow-up records of patients at the out-patient clinic and oncology referrals. The out-patient and emergency hospital records helped us to identify cases who died and those with recurrence.

Diagnosis of recurrence

In most cases, recurrence was considered when there was either an unequivocal increase in fluid-attenuated inversion recovery (FLAIR)/T2 signal abnormality or newly detected areas of contrast enhancement on follow-up MRI requiring further surgery, radiation, or chemotherapy.

Preoperative imaging

**Figure 2:**
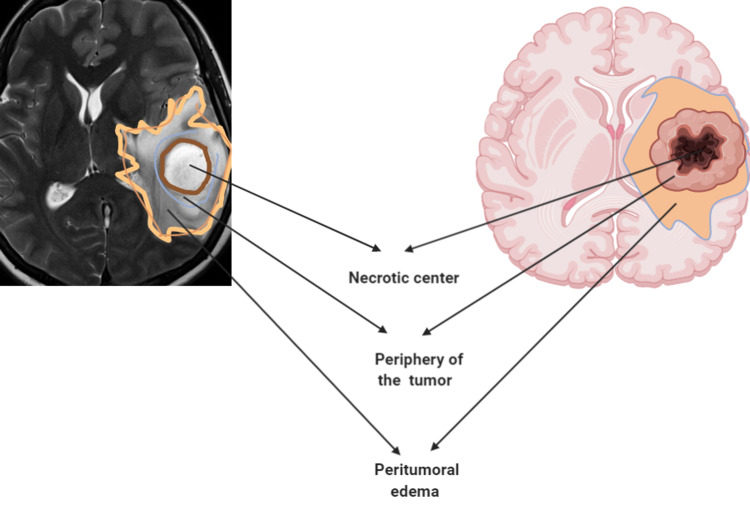
Different GBM regions that were sampled intraoperatively. Created with BioRender.com

Patients who fulfilled all the inclusion criteria underwent preoperative conventional gadolinium-enhanced MRI brain and DCE-MRI. We identified three regions of interest for further analysis to extract qualitative vascular markers. We divided the tumour into a necrotic centre, a peripheral thickness of the tumour, and a peritumoral oedema region (Figure 2). All images were uploaded to the intraoperative navigation system for patients who were defined for surgery.

DCE-MRI data extraction

At first, standard preoperative non-contrasted MRI was obtained, and then, the lesion of interest was identified. Utilizing a fast 3-dimensional gradient echo, the dynamic perfusion MRI was obtained on the 1.5T Philips machine. About 0.1 mmol/kg of gadolinium particles (Omniscan) were injected over 4 seconds through an 18-gauge catheter into a prominent vein using a power injector.

**Figure 3:**
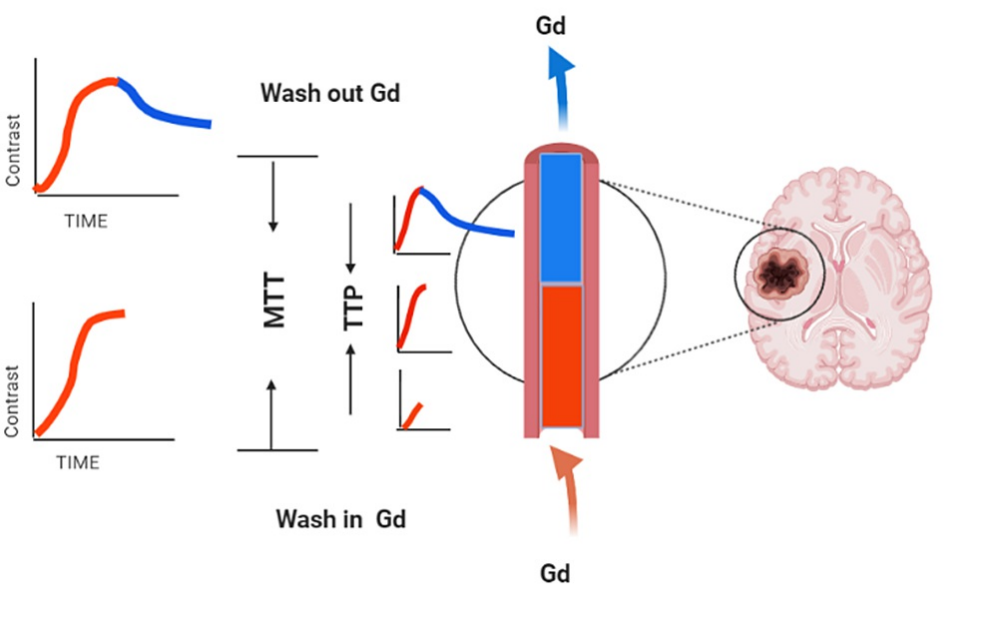
Semi-quantitative parameters of DCE-MRI. Created with BioRender.com

Semi-quantitative parameters are more manageable and more straightforward than quantitative parameters using the T1-weighted sequence after gadolinium injection. We defined specific parameters to extract in a region of interest about 5 mm in diameter. T0 was considered as the basal contrast at a duration of 0 seconds. Time to peak (TTP) was defined as the beginning of contrast injection to the highest enhancement peak. Wash-in is the time of contrast rush in the capillary from zero to peak level, while the washout rate of the contrast is the time elapsed from the peak to the return to zero level (Figure 3).

Surgery

Surgery was done at a maximum of two days post-imaging. Contrasted MRI was loaded into the navigation system to target the predefined areas we specified for sampling. Sampling was done before completely resecting the lesion to avoid the volume shift from debulking and the resulting brain relaxation. The first sample was sent for frozen section pathology. According to the WHO classification 2016 [15,16] and VEGF staining, region-specific samples were sent for histopathological examination.

Immunohistochemistry

All slides were examined under a 200X magnification microscope and scored by a neuropathologist blinded to the tumour, the imaging, and the patient's data. All samples retrieved were labelled according to the region from where they were obtained. All were stained with VEGF and scored from zero to four based on the proportion of cells stained.

Statistical methods and data analysis

All the data were analyzed using R software version 4.0.2 and two-sided, considering P-value ≤ 0.05 was considered statistically significant for all tests. Categorical variables were represented as frequencies and percentages with Chi-square test (or Fisher's exact test, as appropriate) for testing the difference, according to their living/death status. Following the normal distribution test, continuous variables were expressed as means, and standard deviations (SDs) with independent t-test (or Mann-Whitney Test, as appropriate) were used for testing the difference, according to their living/death status.

Pearson's correlation coefficient (r) was used to determine the relationship between different variables and their recurrence correlation. Moreover, univariable and multivariable Cox proportional hazard regression models were used to identify all possible prognostic factors affecting GBM recurrence. The adjustment in the multivariate model was made for other co-founders; TTP (all sites), washout rate (other than periphery), VEGF (all sites), radiotherapy status, and chemo-therapy status of the included patients. Regression results were expressed as hazard ratios (HRs) and 95% confidence interval (95% CI) for both regression models. Kaplan-Meier analysis in the form of survival curves was used to present the recurrence probabilities of significant predictors, and a log-rank test was used to compare their recurrence rates.

## Results

Patients’ clinical data

Two hundred sixty individuals were included in the final analysis, with 180 dead ones and 80 survivors. Radio- and chemo-therapy were received by all surviving patients, and 77.8% (n= 140) of the dead ones. In total, recurrence was confirmed in 69.2% (n= 180) of the patients; seven patients have passed away (Table 1).

**Table 1:** Summary of all parameters stratified by survival outcome. TTP: time to peak, VEGF: vascular endothelial growth factor

Parameter		Dead		Alive		Total		
		n= 180		n= 80		N= 260		
		Mean	Standard Deviation	Mean	Standard Deviation	Mean	Standard Deviation	
TTP center (seconds)		52.5	31.1	44.3	24.6	50.0	28.5	
TTP periphery (seconds)		3.2	2.0	2.3	0.8	2.9	1.8	
TTP peritumoral (seconds)		79.7	14.6	53.7	31.9	71.7	23.5	
Washout rate center (seconds)		54.1	26.5	50.7	35.7	53.1	28.1	
Washout rate periphery (seconds)		24.3	21.4	39.9	34.3	29.1	25.6	
Wash out rate peritumoral (seconds)		73.4	23.3	63.3	28.0	70.3	24.1	
VEGF center (%)		12.2	4.6	10.9	5.9	11.8	4.8	
VEGF periphery (%)		6.4	2.6	5.9	3.6	6.3	2.8	
VEGF peritumoral (%)		17.2	3.5	11.7	7.8	15.5	5.5	
Duration till the first recurrence (months)		8.4	9.0	9.8	11.8	8.8	9.5	
Parameter		n	%	n	%	n	%	
Recurrence	No	40	22.2	40	50.0	80	30.8	
	Yes	140	77.8	40	50.0	180	69.2	
Received Radiotherapy and chemotherapy	No	40	22.2	0	0.0	40	15.4	
	Yes	140	77.8	80	100.0	220	84.6	

Radiological markers of vascularity and immunohistochemistry

The mean TTP, in seconds, was longest at the peritumoral oedema region (71.7±23.5), followed by the tumour's necrotic centre (50.0±28.5) and its periphery (2.9±1.8). The same pattern was observed in the measured washout rates (seconds), where they were high at the peritumoral oedema region (70.3±24.1) and the tumour's centre (53.1±28.1) compared to its periphery (29.1±25.6) (Table 1).

In the same context, the immunohistochemical analysis indicated that VEGF is expressed not only in GBM tissue but also in the peritumoral oedema region. Furthermore, the mean percentage of VEGF positive cells was significantly higher in the peritumoral oedema region (15.5±5.5) and tumour's centre (11.8±4.8), compared with that noted in the surrounding peripheries (6.3±2.8). For all tested parameters, there were no statistically significant differences based on the survival status (Table 1).

Correlation of different biomarkers with recurrence

The expression of VEGF at the peritumoral edema region was inversely correlated to the washout rate at the periphery (r= -0.66; P-value= 0.014) and positively correlated to peritumoral TTP (r= 0.94; P-value< 0.001). Additionally, VEGF expression at the tumor’s center was positively correlated to central TTP (r= 0.89; P-value< 0.001) and the central wash out rate was inversely correlated to TTP at tumor’s periphery (r= -0.56; P-value= 0.045) (Table 2).

**Table 2:** Correlation matrix of different outcomes. TTP: time to peak, VEGF: vascular endothelial growth factor

Variables	Correlation	TTP center	TTP peritumoral	TTP periphery	washout rate center	wash out rate peritumoral	washout rate periphery	VEGF center	VEGF peritumoral	VEGF periphery	Time to recurrence	Survival	Recurrence	Received Radiotherapy and chemo
TTP center	Pearson's r	—												
	P-value	—												
TTP peritumoral	Pearson's r	0.30*	—											
	P-value	0.317*	—											
TTP periphery	Pearson's r	0.25*	0.28*	—										
	P-value	0.403*	0.354*	—										
washout rate center	Pearson's r	0.03*	-0.49	-0.56*	—									
	P-value	0.924*	0.091*	0.045*	—									
wash out rate peritumoral	Pearson's r	0.02*	-0.32*	-0.29	0.74*	—								
	P-value	0.944*	0.282*	0.338*	0.004**	—								
washout rate periphery	Pearson's r	-0.28*	-0.49	-0.11	0.42**	0.45*	—							
	P-value	0.349*	0.087*	0.731*	0.155*	0.121	—							
VEGF center	Pearson's r	0.89*	0.47*	0.25	-0.2	-0.09	-0.5*	—						
	P-value	0.001*	0.106	0.411*	0.504**	0.773**	0.081	—						
VEGF peritumoral	Pearson's r	0.54*	0.94*	0.29*	-0.41	-0.29	-0.66*	0.69**	—					
	P-value	0.057	0.001*	0.336*	0.164*	0.34*	0.014*	0.009**	—					
VEGF periphery	Pearson's r	0.28*	0.51*	-0.08	-0.35*	0.06	-0.55*	0.56*	0.6*	—				
	P-value	0.352*	0.075*	0.795*	0.248	0.845*	0.052*	0.044*	0.031*	—				
Duration till the first recurrence	Pearson's r	0.23*	-0.33*	0.39*	-0.23	-0.18	-0.33*	0.28*	-0.12*	0.05	—			
	P-value	0.443*	0.274*	0.191	0.447*	0.567*	0.268*	0.356*	0.691**	0.883*	—			
Survival	Pearson's r	-0.14*	-0.53*	-0.24*	-0.06	-0.2	0.29	-0.13	-0.48*	-0.1*	0.07**	—		
	P-value	0.651*	0.061	0.42*	0.849**	0.51*	0.333**	0.679**	0.095	0.741**	0.83*	—		
Recurrence	Pearson's r	0.21*	0.16	0.47*	-0.42	-0.41*	-0.64	0.23*	0.32*	0.12	0.65*	-0.28*	—	
	P-value	0.487	0.603*	0.104	0.155*	0.162*	0.018*	0.447*	0.294*	0.695*	0.016*	0.358*	—	
Received Radiotherapy and chemo	Pearson's r	0.4*	-0.22	-0.04*	0.15	-0.08	0.15	0.24*	-0.06	-0.22*	0.34*	0.28*	0.18*	—
	P-value	0.176	0.478*	0.885*	0.623***	0.789**	0.620**	0.424*	0.834*	0.471*	0.249*	0.347*	0.561**	—

**Figure 4:**
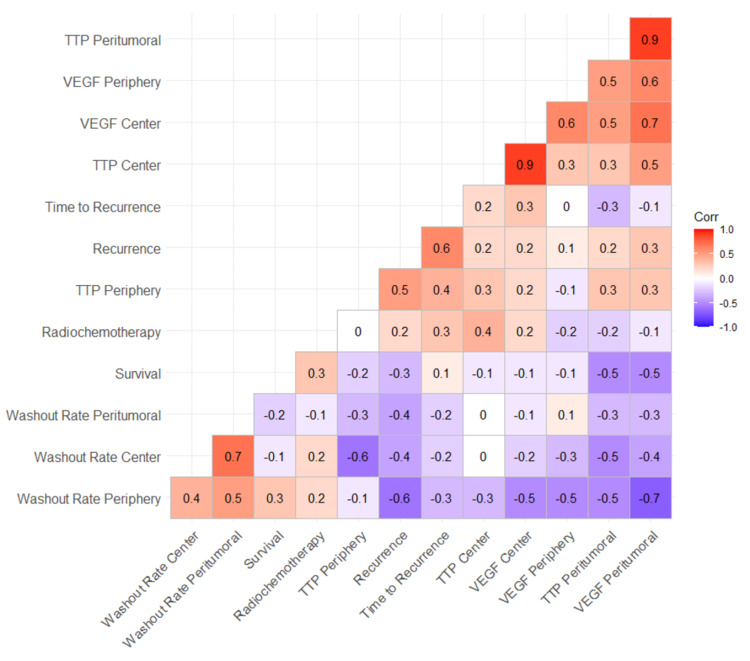
Correlogram with scatterplot matrix. TTP: time to peak, VEGF: vascular endothelial growth factor

The risk of recurrence was solely correlated to the washout rate at the tumor’s periphery (r= -0.64; P-value= 0.018). In contrast, risk of recurrence was not correlated to central TTP (r= 0.21; P-value= 0.487), peritumoral TTP (r= 0.16; P-value= 0.0498), peripheral TTP (r= 0.47; P-value= 0.049), central washout rate (r= -0.42; P-value= 0.0499), peritumoral washout rate (r= -0.41; P-value= 0.044), central VEGF (r= 0.23; P-value= 0.04), peritumoral VEGF (r= 0.32; P-value= 0.049), or peripheral VEGF (r= 0.12; P-value= 0.046) (Figure 4).

**Figure 5:**
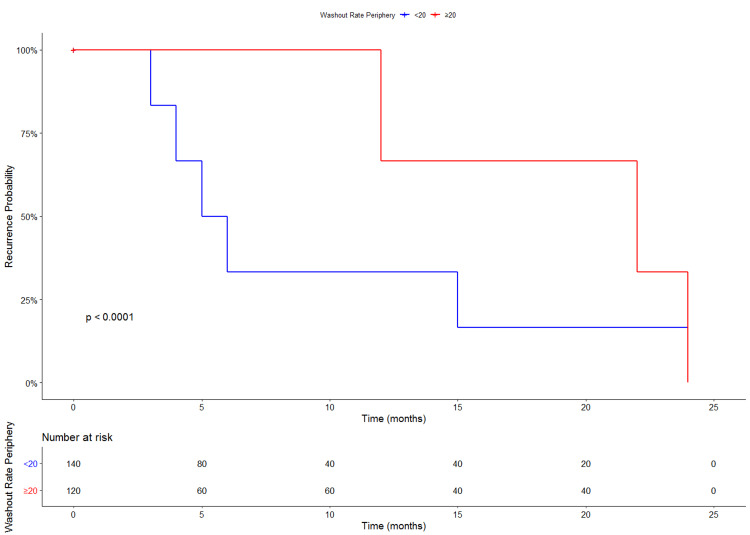
Kaplan-Meier curve for the risk of recurrence according to the washout rate at the periphery.

Although the univariate cox regression did not show a significant increase in recurrence risk with higher washout rate at the tumor’s periphery (HR= 0.99; 95% CI= 0.95-1.04; P-value= 0.05), the multivariate model showed a highly significant association with recurrence risk (HR= 9.08; 95% CI= 8.44-9.76; P-value= 0.05). To test the effect of different peripheral washout rates, 20 seconds cut-off was used. The Kaplan-Meier analysis showed statistically significant results (Figure 5).

## Discussion

Over the last few years, the DCE-MRI has been introduced as a part of the preoperative assessment and the follow-up of brain tumours, including GBM [17,18]. The DCE signal intensity-time curve reflects the combined microvessel permeability, tissue perfusion, and extravascular-space. Therefore, it could be used for a multiparametric characterization of the microvasculature of tumours [19,20]. The advantages of DCE over dynamic susceptibility contrast (DSC)-MRI include the lower possibility of artefacts and the ability to assess the blood-brain barrier (BBB) integrity [20]. Initially, the main focus of DCE metrics was the volume transfer constant (K-trans), which is identified as a permeability marker with BBB disruption and malignant lesions [20-22]. Fitting the DCE data through the two-compartment models, the plasma/ fractional volume of the intravascular compartment was also assessed as a marker for tumour neoangiogenesis and subsequent grading [23,24]. Also, the plasma/fractional volume of the extravascular-extracellular space was evaluated as a candidate marker of mitotic activity [25]. The DSC-MRI is superior to the DCE-MRI for better temporal resolution, with a more accurate blood volume estimation, making it valuable in GBM grading [17].

To date, several studies reported a comparable accuracy of DSC-MRI and DCE-MRI in tumour grading [26-29], with a special focus on DCE-derived K-trans. In a multi-centre study of 94 patients, plasma and extravascular-extracellular space volumes had the highest accuracy for glioma grading [29]. An in-vivo study showed a higher wash-in (p= 0.016) and wash-out (p= 0.014) rates in GBM compared to radiation necrosis [30]. Moreover, TTP was significantly lower in GBM compared to radiation necrosis [30]. In another study of 45 patients, the VEGF expression was correlated with peripheral oedema, enhancement percentage, and the tumour's maximum diameter [31]. The same study found that the peripheral oedema index, enhancement percentage, and the maximum diameter of the tumour were significantly higher in the high-grade than the low-grade tumors; therefore, VEGF could be used as a biomarker for glioma invasion [32].

In this study, we obtained DCE-MRI and conventional MRI for all patients with suspected GBM before surgery. We also obtained frameless navigation guided biopsies from different tumour regions as illustrated before, from the centre, tumour edge and peritumoral tissue guided by the increased T2 and FLAIR intensity surrounding the tumour. Localization was a rough estimate of these regions. There are no strict borders between these regions, resulting in a limitation to precisely sample the theoretical partitions of the GBM. Sampling was attempted before debulking surgery to avoid architectural changes resulting from fluid aspiration or volumetric changes. Each sample was analyzed for the degree of vascular hyperplasia, using VEGF staining. A group of researchers used imaging to sample different tumour regions, and found a correlation between histological markers and certain imaging markers. They observed that the semi-quantitative markers of DCE-MRI were correlated with the enhanced regions, while diffusion parameters were correlated with the non-enhanced regions [16].

**Figure 6:**
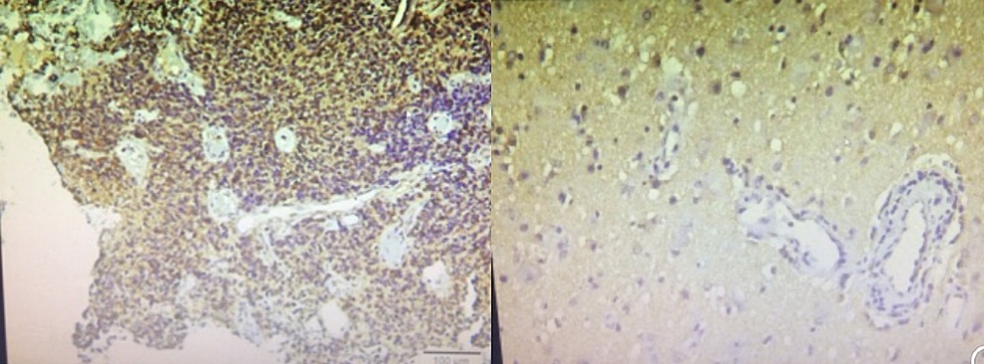
VEGF expression from the area of peritumoral oedema showing high vascular density. VEGF: vascular endothelial growth factor

We found that specific semi-quantitative DCE-MRI markers as the washout rate and TTP were high in the peritumoral oedema area, which indicates the profuse vascularity of that area. Moreover, we found that VEGF expression is high in the peritumoral area and strongly correlates with the washout rate and TTP in the same areas. VEGF expression was higher in the peritumoral area than the centre and tumour edge (Figure 6). At the necrotic centre of the tumour, we observed that VEGF expression was significantly correlated to TTP and microvascular density. We did not observe any correlation of the vascular radiological or histological markers with tumour recurrence except for the washout rate at the tumour's growing edge. The recurrence risk was not correlated with the washout rate at the centre or the peritumoral oedema region.

The observed results may help us to predict vascularity in different regions of the single GBM lesion. That may guide us to target anti-vascular therapy to the highly vascular regions compared to the low vascular regions. Moreover, preoperative non-invasive determination of the microvascularity may aid in prognosis and follow-up (Figure 6).

## Conclusions

Using DCE-MRI, VEGF expression may be used as a non-invasive marker to evaluate tumour grading for clinical prognosis and therapy. Moreover, the risk of GBM recurrence could be determined by evaluating the washout rate at the tumour's periphery. Further large-scale studies are suggested to validate the results and obtain concrete evidence.
